# Importance of the gonococcal MtrCDE efflux pump system in decreasing bacterial susceptibility to zoliflodacin: implications for future clinical treatment of gonorrhea

**DOI:** 10.1128/mbio.01943-26

**Published:** 2026-09-08

**Authors:** Jacqueline T. Balthazar, Concerta L. Holley, Debayan Dey, Graeme L. Conn, William M. Shafer

**Affiliations:** 1 Department of Microbiology and Immunology, Emory University School of Medicine 12239, Atlanta, Georgia, USA; 2 Department of Biochemistry, Emory University School of Medicine 12239, Atlanta, Georgia, USA; 3 The Emory Antibiotic Resistance Center, Emory University School of Medicine 12239, Atlanta, Georgia, USA; 4 Laboratories of Bacterial Pathogenesis, Atlanta Veterans Affairs, Decatur, Georgia, USA; Universiteit Gent, Gent, Belgium

**Keywords:** gonorrhea, antibiotic resistance, zoliflodacin, DNA gyrase, drug efflux, gene regulation

## Abstract

The continued emergence and spread of *Neisseria gonorrhoeae* strains expressing clinical resistance to ceftriaxone (CRO) have stimulated the discovery of new antibiotics to treat gonococcal infections. As the MtrCDE efflux pump system has been linked to the ability of gonococci to develop resistance to multiple antibiotics (e.g., beta-lactams and macrolides), we studied whether it could diminish gonococcal susceptibility to a recently Food and Drug Administration (FDA)-approved antibiotic (zoliflodacin [ZOL]). ZOL recognizes DNA gyrase differently from other antibiotics and will likely be used in the future to treat uncomplicated urogenital tract infections that are refractory to cure by CRO. Herein, we provide evidence that mutations impacting *mtrCDE* gene expression or MtrCDE efflux activity can decrease gonococcal ZOL susceptibility. Moreover, these activities add to resistance levels afforded by a resistance mutation in *gyrB*. Hence, the accumulation of mutations in *gyrB* and *mtr* genes may negatively impact the future clinical efficacy of ZOL.

**IMPORTANCE:**

The newly Food and Drug Administration (FDA)-approved antibiotic zoliflodacin (ZOL) will likely soon be used worldwide to treat gonococcal infections that are refractory to cure with the sole remaining antibiotic used in empiric monotherapy, ceftriaxone (CRO). Here, we find that the level of gonococcal susceptibility to ZOL is linked to expression and function of the MtrCDE efflux pump. This suggests that the future use of this new antibiotic will be challenged by mutations that are already present in clinical strains, as well as by mutations in a ZOL target gene (*gyrB*). Based on our collective observations and to extend the duration of ZOL in treating gonorrhea, we propose that future work should be devoted to developing adjunctive therapies that diminish its export by the MtrCDE efflux pump system.

## OBSERVATION

History is replete with examples in which the clinical efficacy of a newly introduced antibiotic to treat gonorrhea becomes diminished due to the emergence of *Neisseria gonorrhoeae* strains that express clinical resistance, necessitating a change in recommended treatment regimens (1, 2). Zoliflodacin (ZOL) is a newly Food and Drug Administration (FDA)-approved antibiotic that may have significant use in future uncomplicated urogenital tract gonorrheal treatment regimens, especially to eliminate strains expressing resistance to ceftriaxone (CRO), which is currently the sole remaining antibiotic used in empiric monotherapy of gonorrheal infections (3, 4). ZOL is a first-in-class spiropyrimidinetrione that inhibits bacterial type II topoisomerases primarily through interaction with the GyrB subunit of DNA gyrase and to a lesser extent with ParE of topoisomerase IV (3). ZOL had excellent activity against a large panel of gonococcal multi-antibiotic (including ciprofloxacin)-resistant clinical isolates, with MIC values ranging from ≤0.002 to 0.25 µg/mL (4). An MIC breakpoint of ≥0.5 µg/mL was proposed based on these findings.

Previous work showed that *gyrB* mutations that cause single or double amino acid changes at GyrB positions 429 (D429N), 450 (K450T), 467 (S467N), and 429 + 467 can confer ZOL resistance (3, 5). To learn how decreased gonococcal susceptibility to ZOL might develop with or without *gyrB* mutations, we selected spontaneous mutants of strain FA19 at twice the ZOL MIC; these mutants arose at a frequency of 5 × 10^−8^. From this selection, three colonies were recovered, and two were found to express a fourfold increase in the ZOL MIC (0.125 µg/mL) compared to the parent strain (MIC = 0.03 µg/mL). Whole-genome sequencing of DNA from one spontaneous mutant and the parent revealed differences in 12 different coding sequences (Table S1); the accession numbers for these genome sequences are provided in Table 1. Importantly, the spontaneous mutant had a single nucleotide change in *mtrR* (6) that would result in a premature stop codon and loss of the last 11 amino acids of the MtrR repressor protein. As the terminal region of MtrR is needed for dimer formation necessary for effective DNA binding to the *mtrCDE* promoter and repression of transcription (7), we hypothesized that the truncated MtrR would have a reduced capacity to downregulate *mtrCDE* expression. To directly assess whether upregulation of the *mtrCDE* efflux pump operon can impact ZOL susceptibility, as suggested by the presence of the *mtrR202* mutation in the spontaneous mutant, we asked whether the previously described *mtrR171* promoter mutation (8) would also decrease gonococcal ZOL susceptibility. In contrast to *mtrR202*, *mtrR171* is a single bp deletion in the *mtrR* promoter that abrogates *mtrR* expression and improves the −35 hexamer of the overlapping *mtrCDE* promoter that collectively results in increased transcription of *mtrCDE* and elevated gonococcal resistance to antimicrobials above that seen with *mtrR* coding mutations (8, 9). We found that the presence of the *mtrR171* promoter mutation also decreased gonococcal susceptibility to ZOL (Table 1).

**TABLE 1:** Antibiotic susceptibility of isogenic gonococcal strains

Genotype of FA19 strain^*b*^	MIC (µg/mL)^*a*^	
	ZOL	ERY
Wild type	0.03	0.25
Spontaneous mutant with *mtrR202^c^*	0.125	0.5
*mtrR171*	0.25	1.0
*gyrB429*	0.5	0.25
*mtrR171 gyrB429*	2.0	1.0
*mtrR171 gyrB429 mtrD::kan*	0.03	0.06
*mtrD::kan*	0.0078	0.06^*d*^
*mtrD714* (alanine to glycine)^*e*^	0.03	1.0^*d*^
*mtrD823* (lysine to glutamic acid)	0.06	1.0^*d*^
*mtrD825* (serine to alanine)	0.06	0.5^*d*^

^*a*^ MIC values are taken from two to three independent experiments that used the agar dilution method described previously (10).

^*b*^ All genetic derivatives are in the FA19 strain background. The *gyrB429* PCR amplicon used for transformation was prepared using oligonucleotide primers GyrBqRTF (5′-AAACCGAAGAGCCGCTGAAA-3′) and GyrBR (5′-GGCGGTTTCAGACGGCATG-3′), and the protocol for transformation was described previously (11).

^*c*^ The GenBank accession numbers for the whole-genome sequence of parent strain FA19 and the spontaneous mutant are SAMN60422630 and SAMN60422631, respectively.

^*d*^ Ery MIC value is taken from Lyu et al. (12).

^*e*^ Shown in parentheses is the resulting MtrD amino acid change encoded by the introduced mutant allele.

To learn whether the presence of *mtrR171* would influence gonococcal ZOL susceptibility in a *gyrB* mutant, we transformed isogenic FA19 wild type and *mtrR171* strains with a PCR-amplified *gyrB* product (obtained using *gyrB*-specific primers; Table 1 footnote) containing a missense mutation at codon 429. This mutation results in an aspartic acid (D) to asparagine (N) change in GyrB, causing decreased ZOL susceptibility (MIC = 0.5 µg/mL). While the presence of *gyrB429* in both strains decreased gonococcal ZOL susceptibility (Table 1), the transformant bearing both the *mtrR171* promoter mutation and *gyrB429* had a higher level of resistance, which was fourfold (MIC = 2.0 µg/mL) above the proposed clinical breakpoint (0.5 μg/mL) (4).

**Fig 1:**
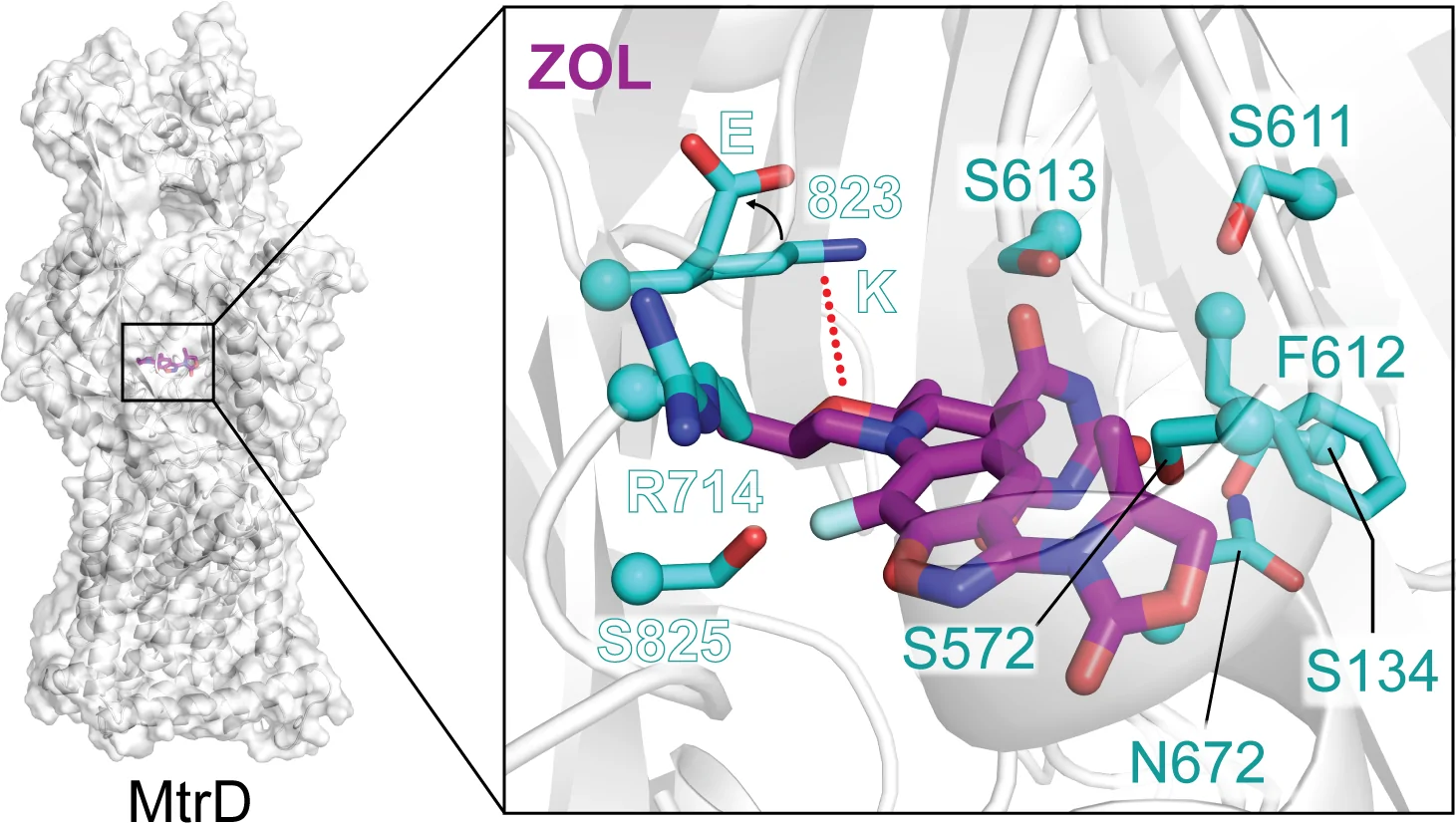
Structural modeling of ZOL binding at the MtrD cleft/entry region. Location of the predicted binding pocket (left) and interactions of ZOL within the cleft/entry opening of MtrD (right). The zoomed view shows both a modeled wild-type (K) or variant (E) residue 823, highlighting the loss of electrostatic contact with the latter. The predicted interaction of ZOL with S825 is also shown. The model is consistent with experimental data showing that changes at MtrD S825 and K823 confer increased ZOL resistance.

As the *mtrR171* promoter mutation with a co-resident *gyrB429* allele had a significant impact on the ZOL MIC, we asked if the loss of the MtrCDE efflux pump would reverse the resistance phenotype. Accordingly, we constructed a transformant of the *mtrR171gyrB429* strain to have with an insertionally inactivated *mtrD* gene*,* which encodes the cytoplasmic membrane protein component of the MtrCDE efflux pump (10, 12–14). A representative *mtrD::kan* transformant was 33-fold more susceptible to ZOL than the parental strain (Table 1), suggesting that, as reported previously (15), ZOL can be exported by the MtrCDE efflux pump. Similarly, the loss of MtrCDE enhanced gonococcal susceptibility to erythromycin (ERY), a known substrate of this pump (10). Earlier work showed that site-directed mutations in *mtrD* that result in single amino acid changes in MtrD retained the ability of the otherwise wild-type gene to alleviate gonococcal hypersusceptibility to certain antimicrobials in an *mtrD::kan* derivative of strain FA19 (12). Further, with some alleles, the level of gonococcal antimicrobial resistance was greater than that afforded by the wild-type gene (12). Using three of the *mtrD* allele variants, we found that, similar to the wild-type gene, replacement of the inactivated *mtrD* by three *mtrD* mutant alleles could reverse ZOL hypersusceptibility in the *mtrD::kan* recipient strain. Importantly, two of the alleles encoding single amino acid changes at codon positions 823 (K to E) and 825 (S to A) rendered the host strain twofold more resistant to ZOL compared to the introduction of the wild-type gene (Table 1). These two amino acids are in the cleft/entry region of MtrD (12, 13) and likely influence the initial binding of ZOL prior to its translocation to the deep binding domain (DBD). The K823E MtrD change was previously shown to enhance gonococcal resistance to macrolides (12). Support for K823E and S825A of MtrD contributing to efflux of ZOL was obtained using *in silico* docking using Glide SP (16, 17), in which the MtrD K823E cryo-EM structure (PDB 8DEW) (12) was used to model ZOL interacting with this transporter. These analyses confirmed the potential interaction of ZOL with K823 and S825 (Fig. 1). We hypothesize that within the cleft/entry region, the K823E change impacts electrostatic interaction with ZOL, while the S825A change influences polar interaction at this site, a mechanism consistent with experimental results showing that these specific MtrD variants confer a twofold increase in ZOL resistance compared to the wild-type transporter.

Our findings have implications for the future success of ZOL as an effective clinical therapeutic option for the treatment of uncomplicated urogenital gonorrhea. Given that mutations that result in the overexpression of the *mtrCDE* operon can decrease gonococcal susceptibility to ZOL and are found in clinical isolates (9), the subsequent emergence of *gyrB* mutations (e.g., *gyrB429*) may significantly decrease ZOL clinical efficacy, especially for pharyngeal infections. Co-resident *mtrD* mutations, which can arise due to horizontal gene transfer from commensal *Neisseria* spp., that result in a mosaic *mtrD* and thus enhanced MtrCDE efflux of antimicrobials (11, 18), could have an additive effect that promotes clinical resistance to ZOL. Notably, the K823E amino acid change in MtrD was found by Ma et al. (19) to be in ca. 10% of gonococcal strains. We posit that mutations that enhance *mtrCDE* expression and missense mutations in *mtrD* that have structural consequences for MtrD-mediated efflux of ZOL will have additive effects with *gyrB* mutations that diminish clinical efficacy of ZOL. To combat this possibility, adjunctive therapeutic approaches, including efflux pump inhibitors, that increase intracellular concentrations of ZOL should be pursued.

## SUPPLEMENTAL MATERIAL

Table S1 mbio.01943-26-s0001.docx Nucleotide changes in mutant.

ASM does not own the copyrights to Supplemental Material that may be linked to, or accessed through, an article. The authors have granted ASM a non-exclusive, world-wide license to publish the Supplemental Material files. Please contact the corresponding author directly for reuse.
