# Supplementary material for: Importance of the gonococcal MtrCDE efflux pump system in decreasing bacterial susceptibility to zoliflodacin: implications for future clinical treatment of gonorrhea
Source: mBio. 2026 Sep 8;17(10):e01943-26. doi: 10.1128/mbio.01943-26 (PMC13712826; doi:10.1128/mbio.01943-26)
Supplement: Supplementary file 1 [file mbio.01943-26-s0001.docx]

| **Table S1. Coding mutations in spontaneous mutant** | | | | | |
| --- | --- | --- | --- | --- | --- |
| **Gene ID^a^** | **Location of the SNP^b^** | **NGO#** | **Gene annotation** | **Nucleotide change^c^** | **Amino Acid Change^d^** |
| VT05_RS16135 | 370022 | NGO_0641 | restriction endonuclease subunit M | ΔTGGC 🡪 TGGC | Reversion of Frameshift |
| VT05_RS15155 | 571842 | NGO_0070 | Porin-like protein, opa54 | T 🡪 G | L180F |
| VT05_RS04505 | 835257 | NGO_1971 | *mafB* | G 🡪 A | S255N |
| VT05_RS05505 | 1021016 | NGO_1285 | *nusA* | C 🡪 G | L322V |
| VT05_RS14825 | 1038675 | NGO_06850 | Hypothetical protein | G 🡪 C | S110T |
| **VT05_RS05955** | **1111504** | **NGO_1366** | ***mtrR*** | **G 🡪 T** | **E202.^e^** |
| VT05_RS06485 | 1214008 | NGO_04980 | Porin-like protein, opa54 | G 🡪 T | L8I |
| VT05_RS06965 | 1317720 | NGO_05420 | Porin-like protein, opa54 | G 🡪 T | G79V |
| VT05_RS07535 | 1404936 | NGO_1158 | Hypothetical protein | T 🡪 C | K128E |
| VT05_RS09700 | 1795743 | NGO_2012 | ABC transporter permease | A 🡪 G | L126P |
| VT05_RS10015 | 1852931 | NGO_11165 | Pilin-like protein, pseudogene | A 🡪 G | Not Determined |
| VT05_RS08235 | 1538128 | NGO_1765 | Glycosyl-transferase-like protein, pseudogene | T 🡪 C | Not Determined |
| ^a^Gene ID in FA19 genome (accession #: NZ_CP012026.1)  ^b^Genomic position of the SNP in FA19 genome  ^c^Nucleotide change in mutant  ^d^Amino acid change in mutant  ^e^Single nucleotide change that results in premature stop codon in *mtrR* | | | | | |
